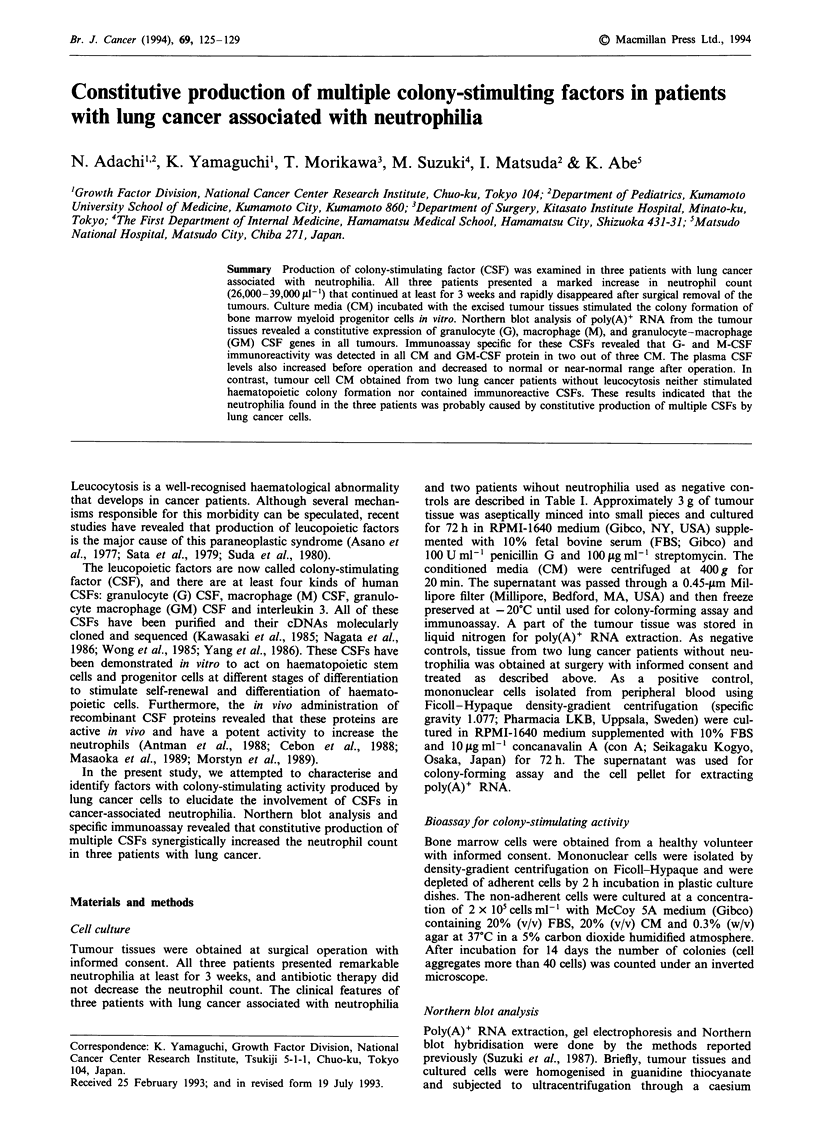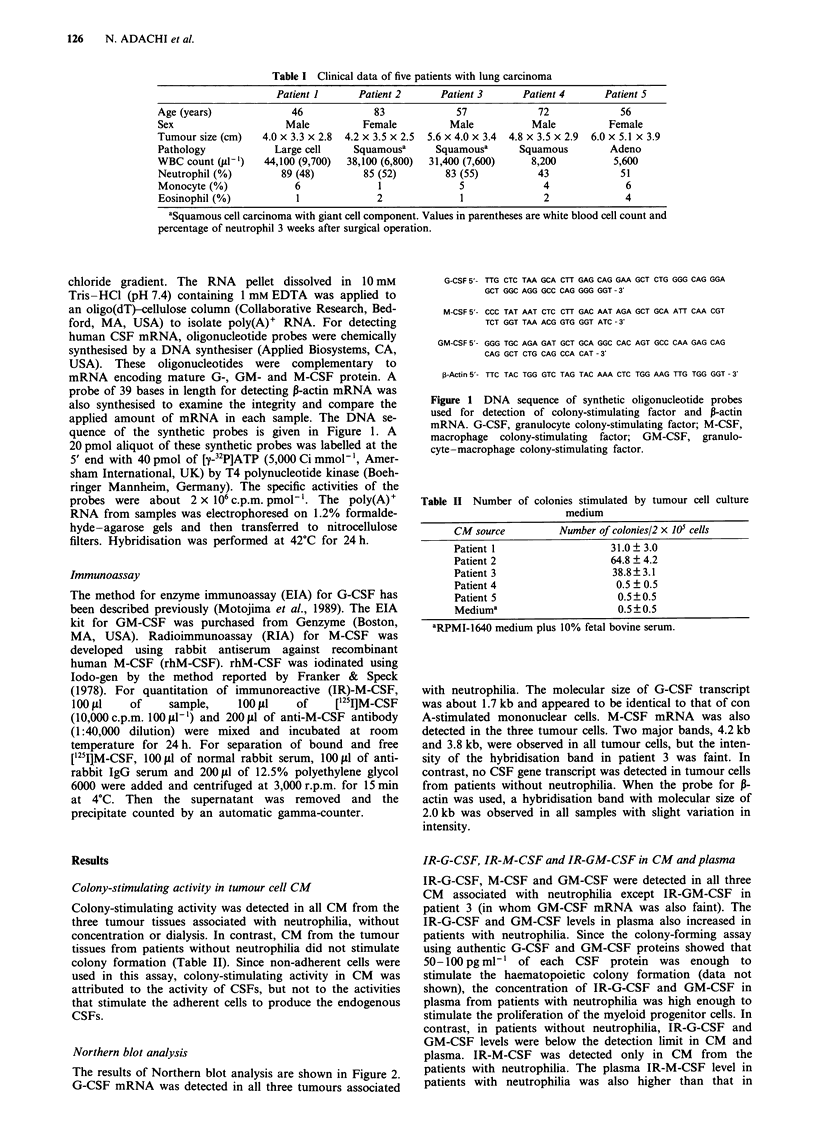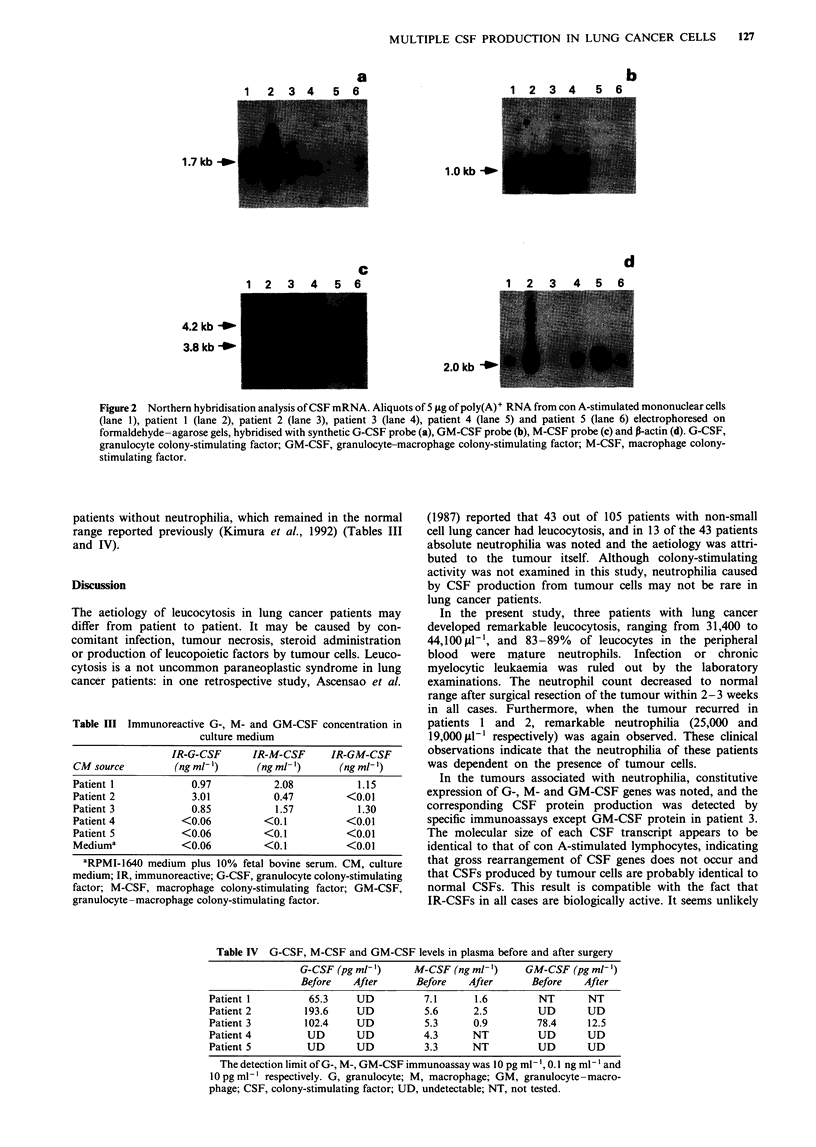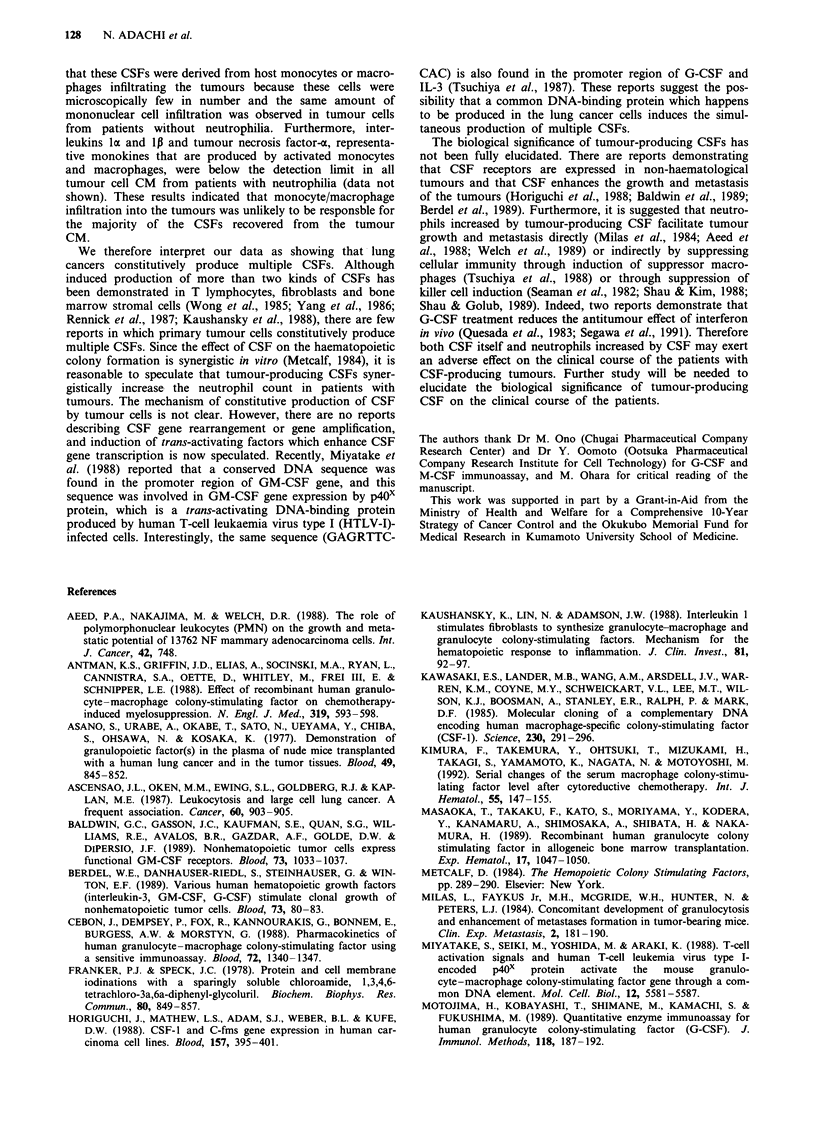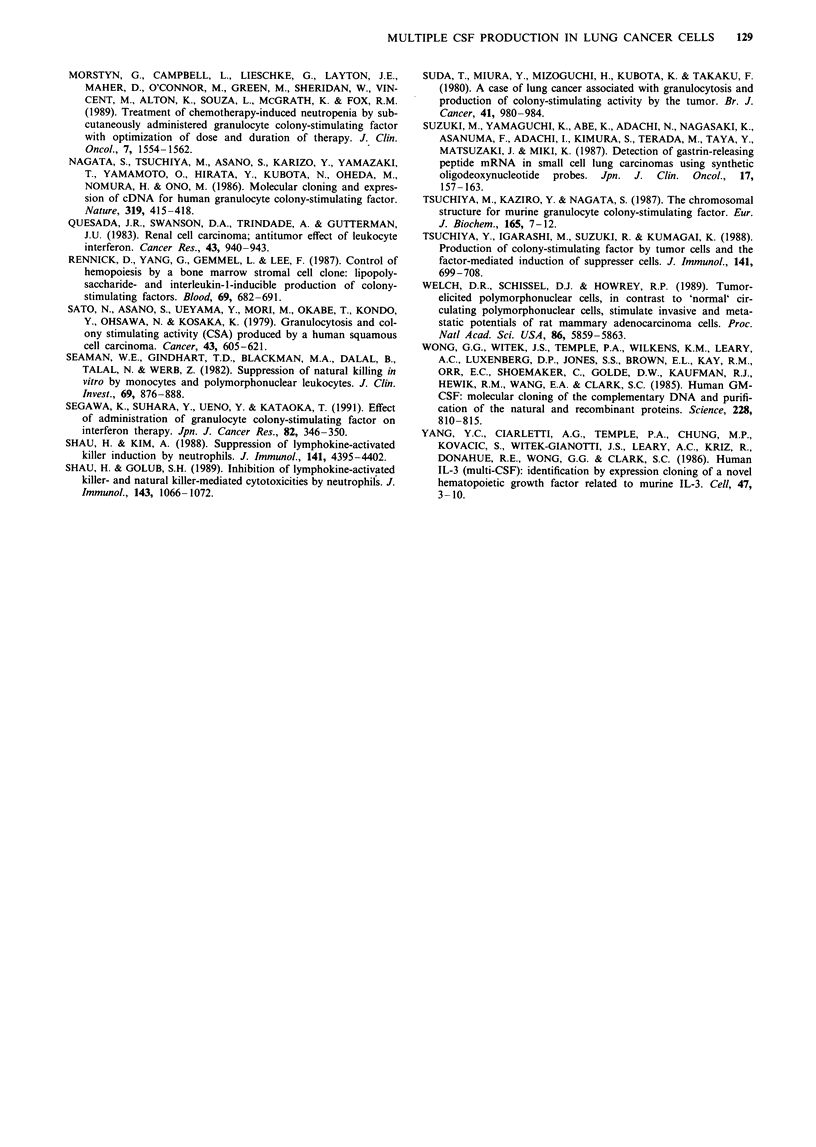# Constitutive production of multiple colony-stimulating factors in patients with lung cancer associated with neutrophilia.

**DOI:** 10.1038/bjc.1994.20

**Published:** 1994-01

**Authors:** N. Adachi, K. Yamaguchi, T. Morikawa, M. Suzuki, I. Matsuda, M. K. Abe

**Affiliations:** Growth Factor Division, National Cancer Center Research Institute, Tokyo, Japan.

## Abstract

**Images:**


Br. J. Cancer (1994), 69, 125-129 © Macmillan Press Ltd., 1994

### Constitutive production of multiple colony-stimulting factors in patients with lung cancer associated with neutrophilia

N. Adachi[1,2], K. Yamaguchi[1], T. Morikawa[3], M. Suzuki[4], I. Matsuda[2] & K. Abe[5]

[1]*Growth Factor Division, National Cancer Center Research Institute, Chuo-ku, Tokyo 104;* [2]*Department of Pediatrics, Kumamoto University School of Medicine, Kumamoto City, Kumamoto 860;* [3]*Department of Surgery, Kitasato Institute Hospital, Minato-ku, Tokyo;* [4]*The First Department of Internal Medicine, Hamamatsu Medical School, Hamamatsu City, Shizuoka 431-31;* [5]*Matsudo National Hospital, Matsudo City, Chiba 271, Japan.*

**Summary** Production of colony-stimulating factor (CSF) was examined in three patients with lung cancer associated with neutrophilia. All three patients presented a marked increase in neutrophil count (26,000-39,000 $\mu l^{-1}$) that continued at least for 3 weeks and rapidly disappeared after surgical removal of the tumours. Culture media (CM) incubated with the excised tumour tissues stimulated the colony formation of bone marrow myeloid progenitor cells *in vitro*. Northern blot analysis of poly(A)$^+$ RNA from the tumour tissues revealed a constitutive expression of granulocyte (G), macrophage (M), and granulocyte-macrophage (GM) CSF genes in all tumours. Immunoassay specific for these CSFs revealed that G- and M-CSF immunoreactivity was detected in all CM and GM-CSF protein in two out of three CM. The plasma CSF levels also increased before operation and decreased to normal or near-normal range after operation. In contrast, tumour cell CM obtained from two lung cancer patients without leucocytosis neither stimulated haematopoietic colony formation nor contained immunoreactive CSFs. These results indicated that the neutrophilia found in the three patients was probably caused by constitutive production of multiple CSFs by lung cancer cells.

Leucocytosis is a well-recognised haematological abnormality that develops in cancer patients. Although several mechanisms responsible for this morbidity can be speculated, recent studies have revealed that production of leucopoietic factors is the major cause of this paraneoplastic syndrome (Asano *et al.*, 1977; Sata *et al.*, 1979; Suda *et al.*, 1980).

The leucopoietic factors are now called colony-stimulating factor (CSF), and there are at least four kinds of human CSFs: granulocyte (G) CSF, macrophage (M) CSF, granulocyte macrophage (GM) CSF and interleukin 3. All of these CSFs have been purified and their cDNAs molecularly cloned and sequenced (Kawasaki *et al.*, 1985; Nagata *et al.*, 1986; Wong *et al.*, 1985; Yang *et al.*, 1986). These CSFs have been demonstrated *in vitro* to act on haematopoietic stem cells and progenitor cells at different stages of differentiation to stimulate self-renewal and differentiation of haematopoietic cells. Furthermore, the *in vivo* administration of recombinant CSF proteins revealed that these proteins are active *in vivo* and have a potent activity to increase the neutrophils (Antman *et al.*, 1988; Cebon *et al.*, 1988; Masaoka *et al.*, 1989; Morstyn *et al.*, 1989).

In the present study, we attempted to characterise and identify factors with colony-stimulating activity produced by lung cancer cells to elucidate the involvement of CSFs in cancer-associated neutrophilia. Northern blot analysis and specific immunoassay revealed that constitutive production of multiple CSFs synergistically increased the neutrophil count in three patients with lung cancer.

### Materials and methods

### Cell culture

Tumour tissues were obtained at surgical operation with informed consent. All three patients presented remarkable neutrophilia at least for 3 weeks, and antibiotic therapy did not decrease the neutrophil count. The clinical features of three patients with lung cancer associated with neutrophilia and two patients wihout neutrophilia used as negative controls are described in Table I. Approximately 3 g of tumour tissue was aseptically minced into small pieces and cultured for 72 h in RPMI-1640 medium (Gibco, NY, USA) supplemented with 10% fetal bovine serum (FBS; Gibco) and 100 U $ml^{-1}$ penicillin G and 100 $\mu g\ ml^{-1}$ streptomycin. The conditioned media (CM) were centrifuged at 400 *g* for 20 min. The supernatant was passed through a 0.45-$\mu m$ Millipore filter (Millipore, Bedford, MA, USA) and then freeze preserved at $-20°C$ until used for colony-forming assay and immunoassay. A part of the tumour tissue was stored in liquid nitrogen for poly(A)$^+$ RNA extraction. As negative controls, tissue from two lung cancer patients without neutrophilia was obtained at surgery with informed consent and treated as described above. As a positive control, mononuclear cells isolated from peripheral blood using Ficoll-Hypaque density-gradient centrifugation (specific gravity 1.077; Pharmacia LKB, Uppsala, Sweden) were cultured in RPMI-1640 medium supplemented with 10% FBS and 10 $\mu g\ ml^{-1}$ concanavalin A (con A; Seikagaku Kogyo, Osaka, Japan) for 72 h. The supernatant was used for colony-forming assay and the cell pellet for extracting poly(A)$^+$ RNA.

### Bioassay for colony-stimulating activity

Bone marrow cells were obtained from a healthy volunteer with informed consent. Mononuclear cells were isolated by density-gradient centrifugation on Ficoll-Hypaque and were depleted of adherent cells by 2 h incubation in plastic culture dishes. The non-adherent cells were cultured at a concentration of $2 \times 10^5$ cells $ml^{-1}$ with McCoy 5A medium (Gibco) containing 20% (v/v) FBS, 20% (v/v) CM and 0.3% (w/v) agar at 37°C in a 5% carbon dioxide humidified atmosphere. After incubation for 14 days the number of colonies (cell aggregates more than 40 cells) was counted under an inverted microscope.

### Northern blot analysis

Poly(A)$^+$ RNA extraction, gel electrophoresis and Northern blot hybridisation were done by the methods reported previously (Suzuki *et al.*, 1987). Briefly, tumour tissues and cultured cells were homogenised in guanidine thiocyanate and subjected to ultracentrifugation through a caesium

Correspondence: K. Yamaguchi, Growth Factor Division, National Cancer Center Research Institute, Tsukiji 5-1-1, Chuo-ku, Tokyo 104, Japan.
Received 25 February 1993; and in revised form 19 July 1993.

**Table I** Clinical data of five patients with lung carcinoma

| | *Patient 1* | *Patient 2* | *Patient 3* | *Patient 4* | *Patient 5* |
|---|---|---|---|---|---|
| Age (years) | 46 | 83 | 57 | 72 | 56 |
| Sex | Male | Female | Male | Male | Female |
| Tumour size (cm) | 4.0 × 3.3 × 2.8 | 4.2 × 3.5 × 2.5 | 5.6 × 4.0 × 3.4 | 4.8 × 3.5 × 2.9 | 6.0 × 5.1 × 3.9 |
| Pathology | Large cell | Squamous[a] | Squamous[a] | Squamous | Adeno |
| WBC count ($\mu l^{-1}$) | 44,100 (9,700) | 38,100 (6,800) | 31,400 (7,600) | 8,200 | 5,600 |
| Neutrophil (%) | 89 (48) | 85 (52) | 83 (55) | 43 | 51 |
| Monocyte (%) | 6 | 1 | 5 | 4 | 6 |
| Eosinophil (%) | 1 | 2 | 1 | 2 | 4 |

[a]Squamous cell carcinoma with giant cell component. Values in parentheses are white blood cell count and percentage of neutrophil 3 weeks after surgical operation.

chloride gradient. The RNA pellet dissolved in 10 mM Tris-HCl (pH 7.4) containing 1 mM EDTA was applied to an oligo(dT)-cellulose column (Collaborative Research, Bedford, MA, USA) to isolate poly(A)$^+$ RNA. For detecting human CSF mRNA, oligonucleotide probes were chemically synthesised by a DNA synthesiser (Applied Biosystems, CA, USA). These oligonucleotides were complementary to mRNA encoding mature G-, GM- and M-CSF protein. A probe of 39 bases in length for detecting β-actin mRNA was also synthesised to examine the integrity and compare the applied amount of mRNA in each sample. The DNA sequence of the synthetic probes is given in Figure 1. A 20 pmol aliquot of these synthetic probes was labelled at the 5′ end with 40 pmol of [γ-$^{32}$P]ATP (5,000 Ci mmol$^{-1}$, Amersham International, UK) by T4 polynucleotide kinase (Boehringer Mannheim, Germany). The specific activities of the probes were about $2 \times 10^6$ c.p.m. pmol$^{-1}$. The poly(A)$^+$ RNA from samples was electrophoresed on 1.2% formaldehyde-agarose gels and then transferred to nitrocellulose filters. Hybridisation was performed at 42°C for 24 h.

*Immunoassay*

The method for enzyme immunoassay (EIA) for G-CSF has been described previously (Motojima *et al.*, 1989). The EIA kit for GM-CSF was purchased from Genzyme (Boston, MA, USA). Radioimmunoassay (RIA) for M-CSF was developed using rabbit antiserum against recombinant human M-CSF (rhM-CSF). rhM-CSF was iodinated using Iodo-gen by the method reported by Franker & Speck (1978). For quantitation of immunoreactive (IR)-M-CSF, 100 μl of sample, 100 μl of [$^{125}$I]M-CSF (10,000 c.p.m. 100 μl$^{-1}$) and 200 μl of anti-M-CSF antibody (1:40,000 dilution) were mixed and incubated at room temperature for 24 h. For separation of bound and free [$^{125}$I]M-CSF, 100 μl of normal rabbit serum, 100 μl of anti-rabbit IgG serum and 200 μl of 12.5% polyethylene glycol 6000 were added and centrifuged at 3,000 r.p.m. for 15 min at 4°C. Then the supernatant was removed and the precipitate counted by an automatic gamma-counter.

G-CSF 5′- TTG CTC TAA GCA CTT GAG CAG GAA GCT CTG GGG CAG GGA GCT GGC AGG GCC CAG GGG GGT - 3′

M-CSF 5′- CCC TAT AAT CTC CTT GAC AAT AGA GCT GCA ATT CAA CGT TCT GGT TAA ACG GTG GGT ATC - 3′

GM-CSF 5′- GGG TGC AGA GAT GCT GCA GGC CAC AGT GCC CAA GAG CAG CAG GCT CTG CAG CCA CAT - 3′

β-Actin 5′- TTC TAC TGG GTC TAG TAC AAA CTC TGG AAG TTG TGG GGT - 3′

**Figure 1** DNA sequence of synthetic oligonucleotide probes used for detection of colony-stimulating factor and β-actin mRNA. G-CSF, granulocyte colony-stimulating factor; M-CSF, macrophage colony-stimulating factor; GM-CSF, granulocyte-macrophage colony-stimulating factor.

**Table II** Number of colonies stimulated by tumour cell culture medium

| *CM source* | *Number of colonies/2 × 10⁵ cells* |
|---|---|
| Patient 1 | 31.0 ± 3.0 |
| Patient 2 | 64.8 ± 4.2 |
| Patient 3 | 38.8 ± 3.1 |
| Patient 4 | 0.5 ± 0.5 |
| Patient 5 | 0.5 ± 0.5 |
| Medium[a] | 0.5 ± 0.5 |

[a]RPMI-1640 medium plus 10% fetal bovine serum.

### Results

*Colony-stimulating activity in tumour cell CM*

Colony-stimulating activity was detected in all CM from the three tumour tissues associated with neutrophilia, without concentration or dialysis. In contrast, CM from the tumour tissues from patients without neutrophilia did not stimulate colony formation (Table II). Since non-adherent cells were used in this assay, colony-stimulating activity in CM was attributed to the activity of CSFs, but not to the activities that stimulate the adherent cells to produce the endogenous CSFs.

*Northern blot analysis*

The results of Northern blot analysis are shown in Figure 2. G-CSF mRNA was detected in all three tumours associated with neutrophilia. The molecular size of G-CSF transcript was about 1.7 kb and appeared to be identical to that of con A-stimulated mononuclear cells. M-CSF mRNA was also detected in the three tumour cells. Two major bands, 4.2 kb and 3.8 kb, were observed in all tumour cells, but the intensity of the hybridisation band in patient 3 was faint. In contrast, no CSF gene transcript was detected in tumour cells from patients without neutrophilia. When the probe for β-actin was used, a hybridisation band with molecular size of 2.0 kb was observed in all samples with slight variation in intensity.

*IR-G-CSF, IR-M-CSF and IR-GM-CSF in CM and plasma*

IR-G-CSF, M-CSF and GM-CSF were detected in all three CM associated with neutrophilia except IR-GM-CSF in patient 3 (in whom GM-CSF mRNA was also faint). The IR-G-CSF and GM-CSF levels in plasma also increased in patients with neutrophilia. Since the colony-forming assay using authentic G-CSF and GM-CSF proteins showed that 50-100 pg ml$^{-1}$ of each CSF protein was enough to stimulate the haematopoietic colony formation (data not shown), the concentration of IR-G-CSF and GM-CSF in plasma from patients with neutrophilia was high enough to stimulate the proliferation of the myeloid progenitor cells. In contrast, in patients without neutrophilia, IR-G-CSF and GM-CSF levels were below the detection limit in CM and plasma. IR-M-CSF was detected only in CM from the patients with neutrophilia. The plasma IR-M-CSF level in patients with neutrophilia was also higher than that in

**Figure 2** Northern hybridisation analysis of CSF mRNA. Aliquots of 5 μg of poly(A)$^+$ RNA from con A-stimulated mononuclear cells (lane 1), patient 1 (lane 2), patient 2 (lane 3), patient 3 (lane 4), patient 4 (lane 5) and patient 5 (lane 6) electrophoresed on formaldehyde-agarose gels, hybridised with synthetic G-CSF probe (**a**), GM-CSF probe (**b**), M-CSF probe (**c**) and β-actin (**d**). G-CSF, granulocyte colony-stimulating factor; GM-CSF, granulocyte-macrophage colony-stimulating factor; M-CSF, macrophage colony-stimulating factor.

patients without neutrophilia, which remained in the normal range reported previously (Kimura *et al.*, 1992) (Tables III and IV).

### Discussion

The aetiology of leucocytosis in lung cancer patients may differ from patient to patient. It may be caused by concomitant infection, tumour necrosis, steroid administration or production of leucopoietic factors by tumour cells. Leucocytosis is a not uncommon paraneoplastic syndrome in lung cancer patients: in one retrospective study, Ascensao *et al.* (1987) reported that 43 out of 105 patients with non-small cell lung cancer had leucocytosis, and in 13 of the 43 patients absolute neutrophilia was noted and the aetiology was attributed to the tumour itself. Although colony-stimulating activity was not examined in this study, neutrophilia caused by CSF production from tumour cells may not be rare in lung cancer patients.

In the present study, three patients with lung cancer developed remarkable leucocytosis, ranging from 31,400 to 44,100 $\mu l^{-1}$, and 83–89% of leucocytes in the peripheral blood were mature neutrophils. Infection or chronic myelocytic leukaemia was ruled out by the laboratory examinations. The neutrophil count decreased to normal range after surgical resection of the tumour within 2–3 weeks in all cases. Furthermore, when the tumour recurred in patients 1 and 2, remarkable neutrophilia (25,000 and 19,000 $\mu l^{-1}$ respectively) was again observed. These clinical observations indicate that the neutrophilia of these patients was dependent on the presence of tumour cells.

In the tumours associated with neutrophilia, constitutive expression of G-, M- and GM-CSF genes was noted, and the corresponding CSF protein production was detected by specific immunoassays except GM-CSF protein in patient 3. The molecular size of each CSF transcript appears to be identical to that of con A-stimulated lymphocytes, indicating that gross rearrangement of CSF genes does not occur and that CSFs produced by tumour cells are probably identical to normal CSFs. This result is compatible with the fact that IR-CSFs in all cases are biologically active. It seems unlikely

**Table III** Immunoreactive G-, M- and GM-CSF concentration in culture medium

| *CM source* | *IR-G-CSF (ng ml$^{-1}$)* | *IR-M-CSF (ng ml$^{-1}$)* | *IR-GM-CSF (ng ml$^{-1}$)* |
|---|---|---|---|
| Patient 1 | 0.97 | 2.08 | 1.15 |
| Patient 2 | 3.01 | 0.47 | <0.01 |
| Patient 3 | 0.85 | 1.57 | 1.30 |
| Patient 4 | <0.06 | <0.1 | <0.01 |
| Patient 5 | <0.06 | <0.1 | <0.01 |
| Medium[a] | <0.06 | <0.1 | <0.01 |

[a]RPMI-1640 medium plus 10% fetal bovine serum. CM, culture medium; IR, immunoreactive; G-CSF, granulocyte colony-stimulating factor; M-CSF, macrophage colony-stimulating factor; GM-CSF, granulocyte-macrophage colony-stimulating factor.

**Table IV** G-CSF, M-CSF and GM-CSF levels in plasma before and after surgery

| | *G-CSF (pg ml$^{-1}$)* | | *M-CSF (ng ml$^{-1}$)* | | *GM-CSF (pg ml$^{-1}$)* | |
|---|---|---|---|---|---|---|
| | *Before* | *After* | *Before* | *After* | *Before* | *After* |
| Patient 1 | 65.3 | UD | 7.1 | 1.6 | NT | NT |
| Patient 2 | 193.6 | UD | 5.6 | 2.5 | UD | UD |
| Patient 3 | 102.4 | UD | 5.3 | 0.9 | 78.4 | 12.5 |
| Patient 4 | UD | UD | 4.3 | NT | UD | UD |
| Patient 5 | UD | UD | 3.3 | NT | UD | UD |

The detection limit of G-, M-, GM-CSF immunoassay was 10 pg ml$^{-1}$, 0.1 ng ml$^{-1}$ and 10 pg ml$^{-1}$ respectively. G, granulocyte; M, macrophage; GM, granulocyte-macrophage; CSF, colony-stimulating factor; UD, undetectable; NT, not tested.

that these CSFs were derived from host monocytes or macrophages infiltrating the tumours because these cells were microscopically few in number and the same amount of mononuclear cell infiltration was observed in tumour cells from patients without neutrophilia. Furthermore, interleukins 1α and 1β and tumour necrosis factor-α, representative monokines that are produced by activated monocytes and macrophages, were below the detection limit in all tumour cell CM from patients with neutrophilia (data not shown). These results indicated that monocyte/macrophage infiltration into the tumours was unlikely to be responsble for the majority of the CSFs recovered from the tumour CM.

We therefore interpret our data as showing that lung cancers constitutively produce multiple CSFs. Although induced production of more than two kinds of CSFs has been demonstrated in T lymphocytes, fibroblasts and bone marrow stromal cells (Wong *et al.*, 1985; Yang *et al.*, 1986; Rennick *et al.*, 1987; Kaushansky *et al.*, 1988), there are few reports in which primary tumour cells constitutively produce multiple CSFs. Since the effect of CSF on the haematopoietic colony formation is synergistic *in vitro* (Metcalf, 1984), it is reasonable to speculate that tumour-producing CSFs synergistically increase the neutrophil count in patients with tumours. The mechanism of constitutive production of CSF by tumour cells is not clear. However, there are no reports describing CSF gene rearrangement or gene amplification, and induction of *trans*-activating factors which enhance CSF gene transcription is now speculated. Recently, Miyatake *et al.* (1988) reported that a conserved DNA sequence was found in the promoter region of GM-CSF gene, and this sequence was involved in GM-CSF gene expression by $p40^X$ protein, which is a *trans*-activating DNA-binding protein produced by human T-cell leukaemia virus type I (HTLV-I)-infected cells. Interestingly, the same sequence (GAGRTTCCAC) is also found in the promoter region of G-CSF and IL-3 (Tsuchiya *et al.*, 1987). These reports suggest the possibility that a common DNA-binding protein which happens to be produced in the lung cancer cells induces the simultaneous production of multiple CSFs.

The biological significance of tumour-producing CSFs has not been fully elucidated. There are reports demonstrating that CSF receptors are expressed in non-haematological tumours and that CSF enhances the growth and metastasis of the tumours (Horiguchi *et al.*, 1988; Baldwin *et al.*, 1989; Berdel *et al.*, 1989). Furthermore, it is suggested that neutrophils increased by tumour-producing CSF facilitate tumour growth and metastasis directly (Milas *et al.*, 1984; Aeed *et al.*, 1988; Welch *et al.*, 1989) or indirectly by suppressing cellular immunity through induction of suppressor macrophages (Tsuchiya *et al.*, 1988) or through suppression of killer cell induction (Seaman *et al.*, 1982; Shau & Kim, 1988; Shau & Golub, 1989). Indeed, two reports demonstrate that G-CSF treatment reduces the antitumour effect of interferon *in vivo* (Quesada *et al.*, 1983; Segawa *et al.*, 1991). Therefore both CSF itself and neutrophils increased by CSF may exert an adverse effect on the clinical course of the patients with CSF-producing tumours. Further study will be needed to elucidate the biological significance of tumour-producing CSF on the clinical course of the patients.

The authors thank Dr M. Ono (Chugai Pharmaceutical Company Research Center) and Dr Y. Oomoto (Ootsuka Pharmaceutical Company Research Institute for Cell Technology) for G-CSF and M-CSF immunoassay, and M. Ohara for critical reading of the manuscript.

This work was supported in part by a Grant-in-Aid from the Ministry of Health and Welfare for a Comprehensive 10-Year Strategy of Cancer Control and the Okukubo Memorial Fund for Medical Research in Kumamoto University School of Medicine.

### References

AEED, P.A., NAKAJIMA, M. & WELCH, D.R. (1988). The role of polymorphonuclear leukocytes (PMN) on the growth and metastatic potential of 13762 NF mammary adenocarcinoma cells. *Int. J. Cancer*, **42**, 748.

ANTMAN, K.S., GRIFFIN, J.D., ELIAS, A., SOCINSKI, M.A., RYAN, L., CANNISTRA, S.A., OETTE, D., WHITLEY, M., FREI III, E. & SCHNIPPER, L.E. (1988). Effect of recombinant human granulocyte-macrophage colony-stimulating factor on chemotherapy-induced myelosuppression. *N. Engl. J. Med.*, **319**, 593-598.

ASANO, S., URABE, A., OKABE, T., SATO, N., UEYAMA, Y., CHIBA, S., OHSAWA, N. & KOSAKA, K. (1977). Demonstration of granulopoietic factor(s) in the plasma of nude mice transplanted with a human lung cancer and in the tumor tissues. *Blood*, **49**, 845-852.

ASCENSAO, J.L., OKEN, M.M., EWING, S.L., GOLDBERG, R.J. & KAPLAN, M.E. (1987). Leukocytosis and large cell lung cancer. A frequent association. *Cancer*, **60**, 903-905.

BALDWIN, G.C., GASSON, J.C., KAUFMAN, S.E., QUAN, S.G., WILLIAMS, R.E., AVALOS, B.R., GAZDAR, A.F., GOLDE, D.W. & DIPERSIO, J.F. (1989). Nonhematopoietic tumor cells express functional GM-CSF receptors. *Blood*, **73**, 1033-1037.

BERDEL, W.E., DANHAUSER-RIEDL, S., STEINHAUSER, G. & WINTON, E.F. (1989). Various human hematopoietic growth factors (interleukin-3, GM-CSF, G-CSF) stimulate clonal growth of nonhematopoietic tumor cells. *Blood*, **73**, 80-83.

CEBON, J., DEMPSEY, P., FOX, R., KANNOURAKIS, G., BONNEM, E., BURGESS, A.W. & MORSTYN, G. (1988). Pharmacokinetics of human granulocyte-macrophage colony-stimulating factor using a sensitive immunoassay. *Blood*, **72**, 1340-1347.

FRANKER, P.J. & SPECK, J.C. (1978). Protein and cell membrane iodinations with a sparingly soluble chloroamide, 1,3,4,6-tetrachloro-3a,6a-diphenyl-glycoluril. *Biochem. Biophys. Res. Commun.*, **80**, 849-857.

HORIGUCHI, J., MATHEW, L.S., ADAM, S.J., WEBER, B.L. & KUFE, D.W. (1988). CSF-1 and C-fms gene expression in human carcinoma cell lines. *Blood*, **157**, 395-401.

KAUSHANSKY, K., LIN, N. & ADAMSON, J.W. (1988). Interleukin 1 stimulates fibroblasts to synthesize granulocyte-macrophage and granulocyte colony-stimulating factors. Mechanism for the hematopoietic response to inflammation. *J. Clin. Invest.*, **81**, 92-97.

KAWASAKI, E.S., LANDER, M.B., WANG, A.M., ARSDELL, J.V., WARREN, K.M., COYNE, M.Y., SCHWEICKART, V.L., LEE, M.T., WILSON, K.J., BOOSMAN, A., STANLEY, E.R., RALPH, P. & MARK, D.F. (1985). Molecular cloning of a complementary DNA encoding human macrophage-specific colony-stimulating factor (CSF-1). *Science*, **230**, 291-296.

KIMURA, F., TAKEMURA, Y., OHTSUKI, T., MIZUKAMI, H., TAKAGI, S., YAMAMOTO, K., NAGATA, N. & MOTOYOSHI, M. (1992). Serial changes of the serum macrophage colony-stimulating factor level after cytoreductive chemotherapy. *Int. J. Hematol.*, **55**, 147-155.

MASAOKA, T., TAKAKU, F., KATO, S., MORIYAMA, Y., KODERA, Y., KANAMARU, A., SHIMOSAKA, A., SHIBATA, H. & NAKAMURA, H. (1989). Recombinant human granulocyte colony stimulating factor in allogeneic bone marrow transplantation. *Exp. Hematol.*, **17**, 1047-1050.

METCALF, D. (1984). *The Hemopoietic Colony Stimulating Factors*, pp. 289-290. Elsevier: New York.

MILAS, L., FAYKUS Jr, M.H., MCGRIDE, W.H., HUNTER, N. & PETERS, L.J. (1984). Concomitant development of granulocytosis and enhancement of metastases formation in tumor-bearing mice. *Clin. Exp. Metastasis*, **2**, 181-190.

MIYATAKE, S., SEIKI, M., YOSHIDA, M. & ARAKI, K. (1988). T-cell activation signals and human T-cell leukemia virus type I-encoded $p40^X$ protein activate the mouse granulocyte-macrophage colony-stimulating factor gene through a common DNA element. *Mol. Cell. Biol.*, **12**, 5581-5587.

MOTOJIMA, H., KOBAYASHI, T., SHIMANE, M., KAMACHI, S. & FUKUSHIMA, M. (1989). Quantitative enzyme immunoassay for human granulocyte colony-stimulating factor (G-CSF). *J. Immunol. Methods*, **118**, 187-192.

MORSTYN, G., CAMPBELL, L., LIESCHKE, G., LAYTON, J.E., MAHER, D., O'CONNOR, M., GREEN, M., SHERIDAN, W., VINCENT, M., ALTON, K., SOUZA, L., MCGRATH, K. & FOX, R.M. (1989). Treatment of chemotherapy-induced neutropenia by subcutaneously administered granulocyte colony-stimulating factor with optimization of dose and duration of therapy. *J. Clin. Oncol.*, **7**, 1554–1562.

NAGATA, S., TSUCHIYA, M., ASANO, S., KARIZO, Y., YAMAZAKI, T., YAMAMOTO, O., HIRATA, Y., KUBOTA, N., OHEDA, M., NOMURA, H. & ONO, M. (1986). Molecular cloning and expression of cDNA for human granulocyte colony-stimulating factor. *Nature*, **319**, 415–418.

QUESADA, J.R., SWANSON, D.A., TRINDADE, A. & GUTTERMAN, J.U. (1983). Renal cell carcinoma; antitumor effect of leukocyte interferon. *Cancer Res.*, **43**, 940–943.

RENNICK, D., YANG, G., GEMMEL, L. & LEE, F. (1987). Control of hemopoiesis by a bone marrow stromal cell clone: lipopolysaccharide- and interleukin-1-inducible production of colony-stimulating factors. *Blood*, **69**, 682–691.

SATO, N., ASANO, S., UEYAMA, Y., MORI, M., OKABE, T., KONDO, Y., OHSAWA, N. & KOSAKA, K. (1979). Granulocytosis and colony stimulating activity (CSA) produced by a human squamous cell carcinoma. *Cancer*, **43**, 605–621.

SEAMAN, W.E., GINDHART, T.D., BLACKMAN, M.A., DALAL, B., TALAL, N. & WERB, Z. (1982). Suppression of natural killing *in vitro* by monocytes and polymorphonuclear leukocytes. *J. Clin. Invest.*, **69**, 876–888.

SEGAWA, K., SUHARA, Y., UENO, Y. & KATAOKA, T. (1991). Effect of administration of granulocyte colony-stimulating factor on interferon therapy. *Jpn. J. Cancer Res.*, **82**, 346–350.

SHAU, H. & KIM, A. (1988). Suppression of lymphokine-activated killer induction by neutrophils. *J. Immunol.*, **141**, 4395–4402.

SHAU, H. & GOLUB, S.H. (1989). Inhibition of lymphokine-activated killer- and natural killer-mediated cytotoxicities by neutrophils. *J. Immunol.*, **143**, 1066–1072.

SUDA, T., MIURA, Y., MIZOGUCHI, H., KUBOTA, K. & TAKAKU, F. (1980). A case of lung cancer associated with granulocytosis and production of colony-stimulating activity by the tumor. *Br. J. Cancer*, **41**, 980–984.

SUZUKI, M., YAMAGUCHI, K., ABE, K., ADACHI, N., NAGASAKI, K., ASANUMA, F., ADACHI, I., KIMURA, S., TERADA, M., TAYA, Y., MATSUZAKI, J. & MIKI, K. (1987). Detection of gastrin-releasing peptide mRNA in small cell lung carcinomas using synthetic oligodeoxynucleotide probes. *Jpn. J. Clin. Oncol.*, **17**, 157–163.

TSUCHIYA, M., KAZIRO, Y. & NAGATA, S. (1987). The chromosomal structure for murine granulocyte colony-stimulating factor. *Eur. J. Biochem.*, **165**, 7–12.

TSUCHIYA, Y., IGARASHI, M., SUZUKI, R. & KUMAGAI, K. (1988). Production of colony-stimulating factor by tumor cells and the factor-mediated induction of suppresser cells. *J. Immunol.*, **141**, 699–708.

WELCH, D.R., SCHISSEL, D.J. & HOWREY, R.P. (1989). Tumor-elicited polymorphonuclear cells, in contrast to 'normal' circulating polymorphonuclear cells, stimulate invasive and metastatic potentials of rat mammary adenocarcinoma cells. *Proc. Natl Acad. Sci. USA*, **86**, 5859–5863.

WONG, G.G., WITEK, J.S., TEMPLE, P.A., WILKENS, K.M., LEARY, A.C., LUXENBERG, D.P., JONES, S.S., BROWN, E.L., KAY, R.M., ORR, E.C., SHOEMAKER, C., GOLDE, D.W., KAUFMAN, R.J., HEWIK, R.M., WANG, E.A. & CLARK, S.C. (1985). Human GM-CSF: molecular cloning of the complementary DNA and purification of the natural and recombinant proteins. *Science*, **228**, 810–815.

YANG, Y.C., CIARLETTI, A.G., TEMPLE, P.A., CHUNG, M.P., KOVACIC, S., WITEK-GIANOTTI, J.S., LEARY, A.C., KRIZ, R., DONAHUE, R.E., WONG, G.G. & CLARK, S.C. (1986). Human IL-3 (multi-CSF): identification by expression cloning of a novel hematopoietic growth factor related to murine IL-3. *Cell*, **47**, 3–10.